# The Reflectance Characteristics of an Inverse Moth-Eye Structure in a Silicon Substrate Depending on SF_6_/O_2_ Plasma Etching Conditions

**DOI:** 10.3390/mi13101556

**Published:** 2022-09-20

**Authors:** Jong-Chang Woo, Doo-Seung Um

**Affiliations:** 1 Department of Semiconductor Process Equipment, Semiconductor Convergence Campus of Korea Polytechnic, Anseong-si 17550, Gyeonggi-do, Korea; 2 Department of Electrical Engineering, Sejong University, Seoul 05006, Korea

**Keywords:** photovoltaic efficiency, plasma etching, surface roughness, reflectance, texturing, inverse moth-eye structure

## Abstract

The global RE100 campaign is attracting attention worldwide due to climate change caused by global warming, increasingly highlighting the efficiency of renewable energy. Texturing of photovoltaic devices increases the devices’ efficiency by reducing light reflectance at their surfaces. This study introduces the change in light reflectance following the process conditions of plasma etching as a texturing process to increase the efficiency of photovoltaic cells. Isotropic etching was induced through plasma using SF_6_ gas, and the etch profile was modulated by adding O_2_ gas to reduce light reflectance. A high etch rate produces high surface roughness, which results in low surface reflectance properties. The inverse moth-eye structure was implemented using a square PR pattern arranged diagonally and showed the minimum reflectance in visible light at a tip spacing of 1 μm. This study can be applied to the development of higher-efficiency optical devices.

## 1. Introduction

A considerable proportion of greenhouse gas emissions that cause global warming are from power plants that use fossil fuels [1,2]. Accordingly, mankind has been seeking alternative energy, also called green energy, with little-to-no greenhouse gas emissions and has been striving to increase the efficiency of energy-consuming devices, such as vehicles and electronic devices [3,4]. Among the many green energy sources, photovoltaic devices can be used in both large-scale facilities and small-scale installations, such as the roof of a house and a vehicle, and thus have a high degree of usability and accessibility [5,6]. Accordingly, various studies on photovoltaic devices are currently being conducted from material and structural perspectives. Among the many materials used for photovoltaic devices, silicon is fascinating due to its abundance in the Earth’s crust, nontoxicity, and high durability [7,8,9]. In addition, the manufacturing process of silicon-based photovoltaic devices has been well-established due to accumulated technical knowledge, as they have been studied and developed for a long time [10,11]. However, the optical loss due to the high light reflectivity of the silicon surface is an obstacle to improving the efficiency of photovoltaic devices [12,13]. To maximize the conversion efficiency of light into electricity, the light loss on the device surface should be minimized [14]. Light reflectance can be reduced by antireflection (AR) coating and surface texturing [15,16]. The surface texturing of silicon substrates has typically resulted in a pyramid-shaped microstructure through wet etching (alkaline) or into a moth-eye or inverse moth-eye structure through plasma etching, nanoimprint, nanoscribe, colloidal lithography, and laser ablation techniques [17,18,19,20,21,22,23,24]. The advantages of wet etching for microstructures are that the process is simple and large-area processing is easy. However, this process is difficult to apply to nanostructures due to the orientation dependence of alkaline etching in silicon wafers and its limitations in selecting other materials for photovoltaic devices. Conversely, plasma etching for moth-eye-inspired structures can be applied to various substrates, and the slope of the microstructure can be controlled using the process parameters [25,26,27]. Moreover, nanostructures can be formed using plasma etching; thus, they can be applied to various devices, such as photodetectors, LED devices, and solar cells [28]. In addition, a photovoltaic device with a moth-eye structure can keep its surface clean due to its super hydrophobic characteristics [29,30,31].

This study investigated the effect of process variables on the surface reflectance of silicon-based inverse moth-eye structures. SF_6_ gas was selected to fabricate the microstructure, and O_2_ gas was added to investigate the change in reflectance by adjusting the etch rate and aspect ratio. In addition, the etch rate and reflectance were investigated by controlling upper RF power and process pressure. To determine the surface characteristics of the etched substrate, the shape and surface roughness of the microstructure were checked using a scanning electron microscope (SEM) and an atomic force microscope (AFM). The reflectance according to the process variables was investigated through UV-Vis spectroscopy.

## 2. Materials and Methods

All samples were used by cutting a (100)-oriented p-type 6-inch silicon wafer (resistivity: 1–30 Ω, single-side polished) into 20 × 20 mm pieces through a dicing machine. Ultrasonic cleaning was performed in isopropyl alcohol for 10 min to remove dust and impurities from the sample surface, followed by rinsing with deionized (DI) water and drying with N_2_, sequentially. The cleaned silicon substrate was dried in a vacuum oven at 80 °C for 5 min to completely remove moisture from the surface.

**Figure 1:**
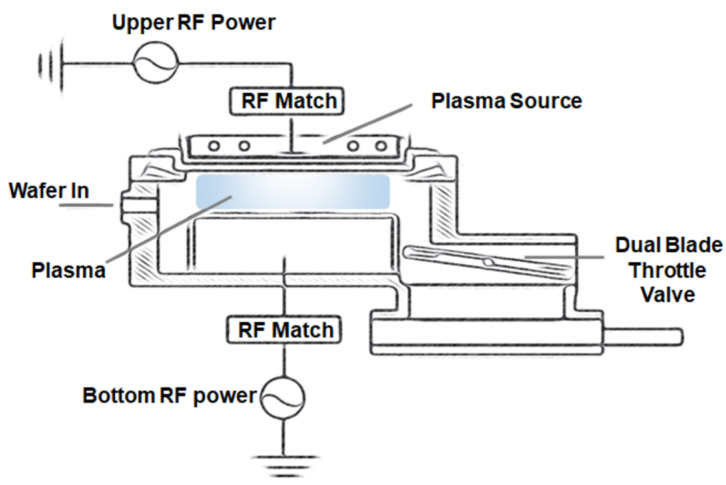
Schematic illustration of the planar HDP system used in this experiment.

Photolithography and plasma etching processes were used to fabricate the inverse moth-eye structure. The photoresist (PR) used PFi-38A (I-line PR, Sumimoto Chemical, Japan) manufactured by Sumimoto Chemical Co., Ltd., and the photomask was self-produced. The I-line (365 nm wavelength) exposure process was performed with stepper equipment (Nikon NSR-2205i11D, Nikon, Japan) and a square-pattern photomask (1 µm width). Hexamethyldisilazane (HMDS) was spin-coated at 4000 rpm for 1 min and then annealed at 110 °C for 1 min to increase the adhesion between PR and the silicon substrate. To achieve a 1.3-µm thickness pattern, PFi-38A PR was spin-coated at 2500 rpm for 1 min and then annealed at 90 °C for 1 min. The samples were exposed to 365 nm UV light for 15 s. Afterward, the sample was developed on the AZ 300MIF developer for 90 s, rinsed in DI water, and dried by N_2_ blowing. Before plasma etching, all samples were annealed on a 90 °C hot plate for 1 min. Figure 1 shows the schematics of the planar high-density plasma (HDP, SELEX 200) system used in this experiment. The HDP system comprises a vacuum chamber, a plasma source that generates magnetic fields to dissociate the injected gas into the plasma, a cathode to control the movement of positive charges in the plasma, and a throttle valve to control the process pressure. The plasma source was designed as a mixed structure of the coil type of an inductively coupled plasma source and the parallel plate type of a capacitively coupled plasma source. The vacuum of the chamber was initially maintained at 5.5 × 10^−5^ Torr using turbo and rotary pumps, and the process pressure was controlled using a throttle valve. Furthermore, the gas flow rate was controlled by a mass flow controller. The upper RF power, process pressure, and amount of O_2_ gas added to 100 sccm of SF_6_ gas were controlled as etching parameters for the inverse moth-eye structure. Meanwhile, the bottom (platen) RF power and substrate temperature were fixed at 50 W and 45 °C, respectively.

The process evaluation and confirmation of the inverse moth-eye structure were investigated using a field-emission scanning electron microscope (FE-SEM, Sirion 400, Cambridge, MA, USA). The surface roughness of the etched silicon substrate was investigated using an AFM (Dimension 3100, Veeco, Plainview, TX, USA), and the surface reflectance was measured using a UV-Vis spectrophotometer (Hitachi U3100, Hitachi, Ibaraki, Japan).

## 3. Results and Discussion

**Figure 2:**
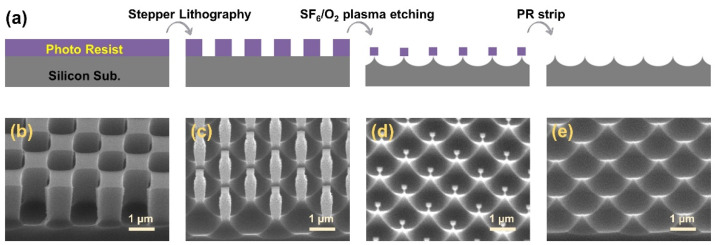
(**a**) The process schematic diagram of an inverse moth-eye microstructure. (**b**–**e**) SEM images according to the process steps: (**b**) grating patterns by the lithography process, etching profile (**c**) at the beginning and (**d**) end of the process depending on etch time, and (**e**) inverse moth-eye structure after PR strip process.

Figure 2a shows a process schematic diagram of the inverse moth-eye microstructure of a silicon substrate. The detailed conditions and materials are described in the experiments section. For the plasma etching process, SF_6_ gas is advantageous for microscale silicon etching because it can form considerable F radicals in the plasma. Plasma etching exhibits anisotropic properties; however, the etching profile would be isotropic if only a chemical gas is used under certain conditions [32,33]. The O radicals formed by the added oxygen gas protect the sidewalls of the etched silicon, which may induce a change in the isotropic etching profile, resulting in a change in the aspect ratio of the sharp cone shape [34,35]. Therefore, the SF_6_/O_2_-based plasma was used to fabricate a microsized pointed inverse moth-eye structure. The diagonally arranged square pattern is formed through the lithography process (Figure 2b). The opened silicon surface is etched in a semielliptical shape following the isotropic etching characteristics in SF_6_/O_2_ plasma. As the etching progresses, the height and width of the square PR patterns decrease, and the etch depth and width of the silicon substrate increase (Figure 2c). As the etching proceeds further, the PR patterns remain small, and the silicon substrate forms an inverse moth-eye structure (Figure 2d). Finally, a sharp cone shape is left on the surface when the remaining PR is removed with acetone (Figure 2e).

**Figure 3:**
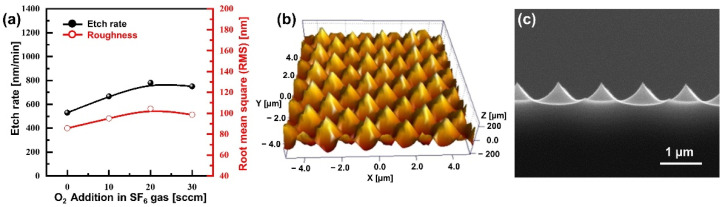
(**a**) Etch rate and surface roughness following the amount of O_2_ gas added to 100 sccm of SF_6_ gas. (**b**) AFM and (**c**) cross-sectional SEM images of silicon substrate etched in SF_6_/O_2_ plasma with 20 sccm O_2_ gas addition.

In general, the surface reflectance decreases as the roughness of the silicon surface increases. Additionally, the surface roughness increases as the etching rate of the patterned substrate increases. That is, the etch rate and the surface reflectance have a negative correlation. Therefore, the etch rate, surface roughness, and surface reflectance need to be investigated to prove this correlation and obtain the minimum surface reflectance. Figure 3a shows the etch rate and surface roughness following the addition of O_2_ gas to 100 sccm of SF_6_ gas under an upper RF power of 850 W, bottom RF power of 50 W, process pressure of 35 mTorr, and a substrate temperature of 45 °C. Under pure SF_6_ plasma, the etch rate of the silicon substrate is about 530 nm/min, and the surface roughness (root mean square, RMS) is 85.74 nm. As the amount of O_2_ gas is increased, the etch rate and surface roughness goes through a maximum and slightly decrease [36,37]. The etch rate and the roughness were at their maximum of 780 nm/min and 104.41 nm, respectively, when 20 sccm of O_2_ gas was added. Figure 3b shows the AFM image after etching with 20 sccm of O_2_ gas and a high roughness with a tip-to-tip distance of 2 µm. The etch rate and surface roughness are slightly decreased to 750 nm/min and 98.54 nm, respectively, when O_2_ gas is further added. Figure 3c is a cross-sectional SEM image of a silicon substrate etched with 20 sccm of O_2_ gas, showing the pointed tips and semielliptical silicon substrate.

**Figure 4:**
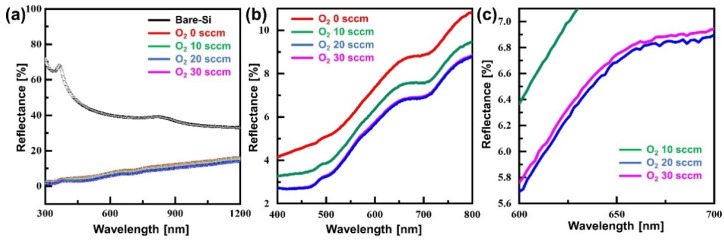
The reflectance spectra of an inverse moth-eye-structured silicon substrate depending on the SF_6_/O_2_ gas mixing ratio: (**a**) wavelength of 300–1200 nm, (**b**) magnified at 400–800 nm wavelength, and (**c**) magnified at 600–700 nm wavelength.

Figure 4 shows the reflectance characteristics of the etched surface following the ratio of the SF_6_/O_2_ gas mixture. The reflectance of the bare silicon substrate shows an average reflectance of 41.9% at wavelengths of 400 to 800 nm. In contrast, all samples etched with inverse moth-eye structures show reflectance characteristics below 10%. As the amount of O_2_ gas is increased to 20 sccm, the surface reflectance decreases. Furthermore, the reflectance of the etched surface slightly decreased when 30 sccm of O_2_ gas was added. In particular, the etched sample at the 100 sccm SF_6_ gas added with 20 sccm O_2_ gas had the minimum surface reflectance at 5.42%. This is correlated with the surface roughness, and this result is consistent with the surface roughness data shown in Figure 3a.

**Figure 5:**
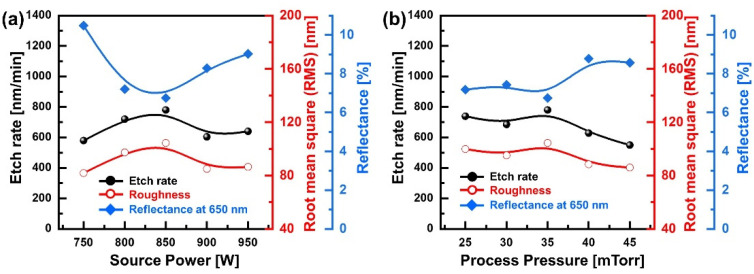
Etch rate, surface roughness, and reflectance following the process conditions: (**a**) source RF power and (**b**) process pressure.

Various variables, such as upper RF power and process pressure, exist in the plasma etching process, and the etch rate and etch profile can be changed by tuning these conditions [38,39]. For example, an increase in upper RF power increases the dissociation action of the injected gas, increasing the density of radicals in the plasma, and affecting the electron temperature in a certain range [40,41]. Moreover, the process pressure is related to the plasma density, electron temperature, and mean free path of electrons and radicals [42,43,44]. This means that the surface roughness and the reflectance can be modulated depending on the process parameters. Therefore, changes in the etch rate, surface roughness, and reflectance were investigated following the upper RF power and process pressure. Figure 5a shows the etch rate, surface roughness, and reflectance following the upper RF power under the conditions of a gas mixing ratio of SF_6_/O_2_ (100 sccm/20 sccm), a bottom RF power of 50 W, and a process pressure of 35 mTorr. As the upper RF power increases from 750 to 850 W, the etch rate and surface roughness increase from 579 to 780 nm/min and 81.78 to 104.41 nm, respectively, and the reflectance at a 650 nm wavelength decreases from 10.48% to 6.75%. However, when the upper RF power is increased above 850 W, the etch rate and surface roughness decrease, and the reflectance increases. Figure 5b shows the etch rate, surface roughness, and reflectance following the process pressure at an SF_6_/O_2_ (100 sccm/20 sccm) gas mixing ratio, an upper RF power of 850 W, and bottom RF power of 50 W. At process pressures below 35 mTorr, the etch rate was maintained between 685 and 780 nm/min without any significant change, and thus, the surface roughness and the reflectance were maintained between 95.14 nm and 104.41 nm and 6.75% and 7.43%, respectively. As the process pressure increases above 35 mTorr, the etch rate and surface roughness significantly decrease, leading to an increase in reflectance. In both process variables, the etch rate and surface roughness show a positive correlation, and the surface roughness and reflectance show a negative correlation.

**Figure 6:**
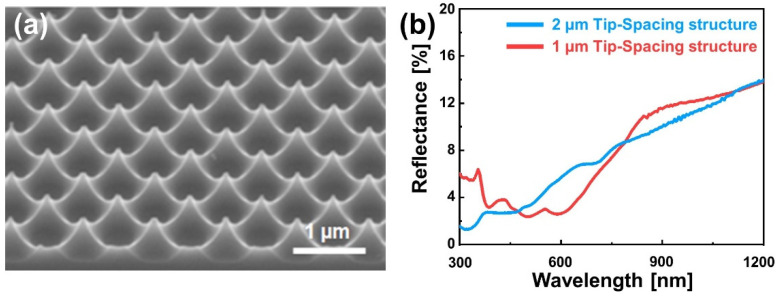
(**a**) Silicon microstructure with a 1-µm tip spacing. (**b**) The reflectance spectra following the density of the microstructure.

Figure 6a shows an inverse moth-eye structure with a short tip spacing (1 µm), in which a diagonally arranged square PR pattern (0.5 µm width) was used. The tips were denser when compared with the structure of a 2-µm tip spacing (Figure 2e). The 1-µm tip-spacing structure has a low reflectance in the visible light region; the reflectance at about 590 nm wavelength is 2.59%, which is significantly decreased from that of the 2-µm tip-spacing structure. Fabricating the sharp tip structure was difficult when a square PR pattern of ≥2 µm was used. This is probably because the depth and width of the silicon substrate to be etched increase as the pattern becomes wider, while all the PR is etched before forming the sharp tip structure due to the limited height of the PR.

## 4. Conclusions

This study investigated the etch rate, surface roughness, and light reflectance following the plasma etching parameters to optimize the silicon-based inverse moth-eye structure. Isotropic etching for inverse moth-eye structures was induced by chemical plasma etching with SF_6_ gas, and the modification of the etching profile was induced by adding O_2_ gas, an etching inhibitor. The inverse moth-eye structure was confirmed through FE-SEM analysis. In this study, the surface change due to the SF_6_/O_2_ gas mixing ratio, upper RF power, and process pressure process conditions were mainly investigated. The etching rate and surface roughness were at a maximum when the SF_6_/O_2_ gas mixture ratio was 100 sccm/20 sccm. Due to this, the reflectance in visible light was observed to be at the minimum. Furthermore, the etch rate and surface roughness were at the maximum and the reflectance was at the minimum at an upper RF power of 850 W between 750 and 950 W. When the process pressure was 35 mTorr, between 25 and 45 mTorr, the etch rate and surface roughness were at the maximum, and the reflectance was at the minimum. Finally, the light reflectance in the visible light region can be confirmed to decrease as the tip spacing of the inverse moth-eye structure decreased from 2 to 1 μm. This study can be expected to provide important process data for improving the efficiency of silicon-based optical devices.

## Data Availability

The data that support the findings of this study are available with in the article and its Supplementary Material.

## Supplementary Materials

The following supporting information can be downloaded at: https://www.mdpi.com/article/10.3390/mi13101556/s1, Figure S1: SEM images of an inverse moth-eye microstructure according to O_2_ gas addition; Figure S2: SEM images of an inverse moth-eye microstructure according to upper RF power; Figure S3: SEM images of an inverse moth-eye microstructure according to process pressure.
