# Supplementary material for: The Reflectance Characteristics of an Inverse Moth-Eye Structure in a Silicon Substrate Depending on SF6/O2 Plasma Etching Conditions
Source: Micromachines (Basel). 2022 Sep 20;13(10):1556. doi: 10.3390/mi13101556 (PMC9607972; doi:10.3390/mi13101556)
Supplement: Supplementary file 1 [file micromachines-13-01556-s001.zip › micromachines-1932787-supplementary.pdf]

micromachines

MDPI

Supplementary Materials

The reflectance characteristics of an inverse moth-eye structure in a silicon substrate depending on SF_6/O_2 plasma etching conditions

Jong-Chang Woo [1], Doo-Seung Um [2,*]

[1] Department of Semiconductor Process Equipment, Semiconductor Convergence Campus of Korea Polytechnic, 41-12, Songwon-gil, Gongdo-eup, Anseong-si, Gyeonggi-do 17550, Republic of Korea
[2] Department of Electrical Engineering, Sejong University, Seoul 05006, Republic of Korea

* Correspondence: dsum@sejong.ac.kr (D. S. U.)

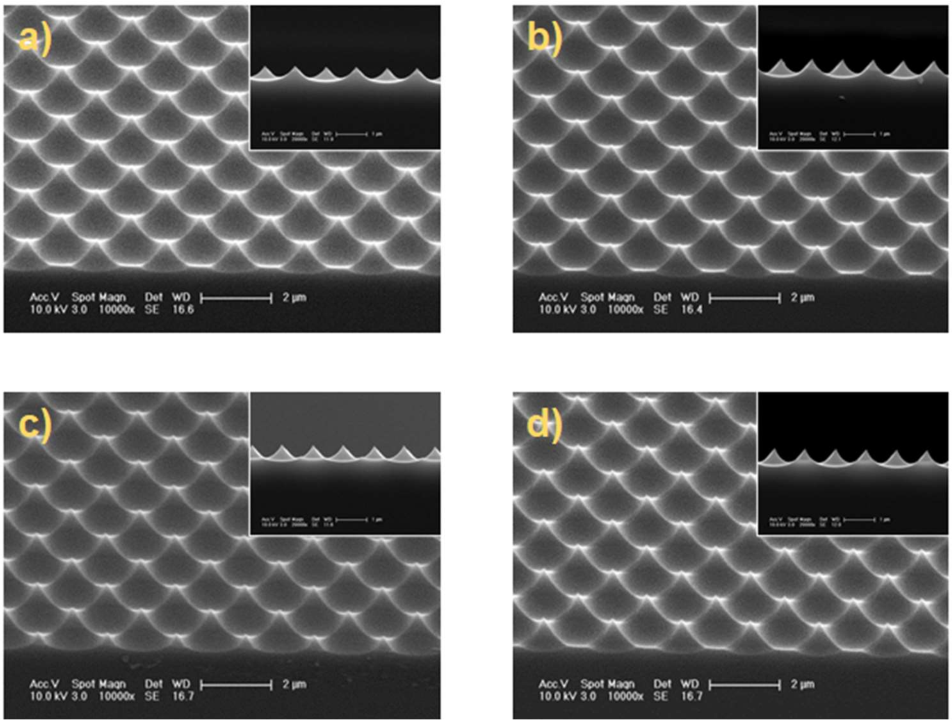

Figure S1. SEM images of an inverse moth-eye microstructure according to O_2 gas addition; (a) 0 sccm, (b) 10 sccm, (c) 20 sccm and (d) 30 sccm

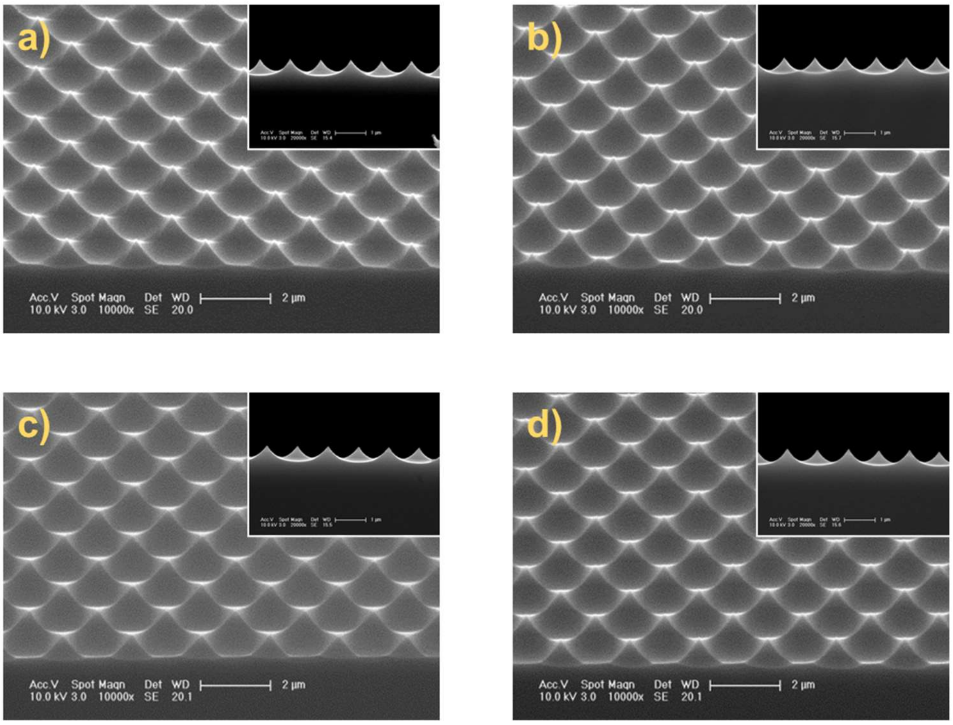

Figure S2. SEM images of an inverse moth-eye microstructure according to upper RF power; (a) 750 W, (b) 800 W, (c) 900 W and (d) 950 W

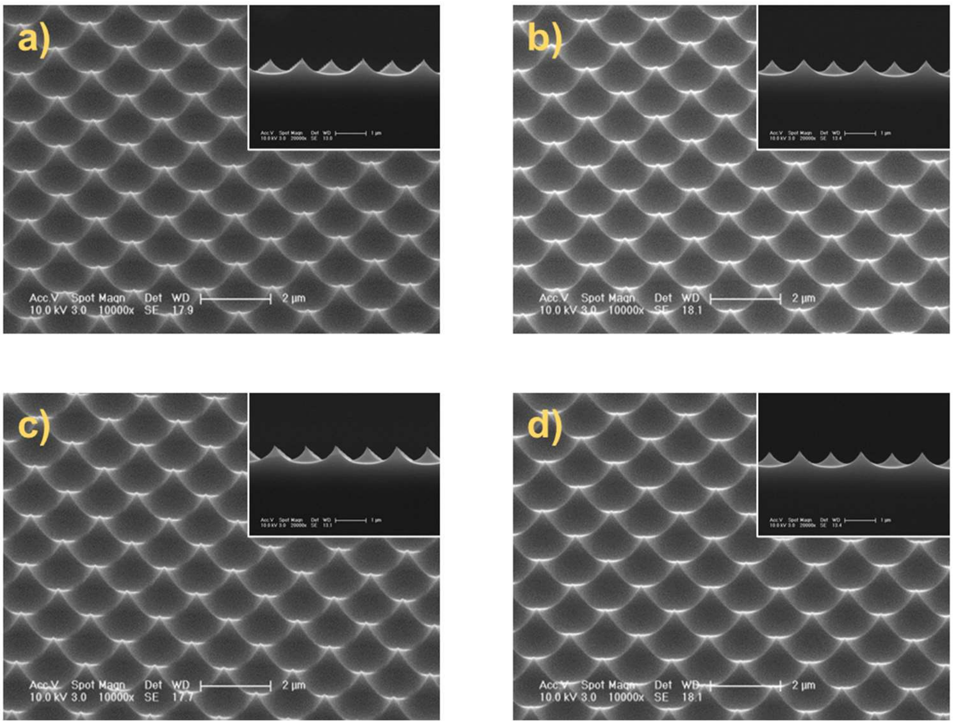

Figure S3. SEM images of an inverse moth-eye microstructure according to process pressure; (a) 25 mTorr, (b) 30 mTorr, (c) 40 mTorr and (d) 45 mTorr
